# (2*E*,6*E*)-2,6-Bis(4-methyl­benzyl­idene)cyclo­hex-3-en-1-one

**DOI:** 10.1107/S1600536811055632

**Published:** 2012-01-11

**Authors:** M. Saeed Abaee, Werner Massa, Mohammad M. Mojtahedi, A. Wahid Mesbah

**Affiliations:** a Organic Chemistry Laboratory, Chemistry and Chemical Engineering, Research Center of Iran, PO Box 14335-186, Tehran, Iran; b Fachbereich Chemie der Philipps-Universität, Hans-Meerwein-Strasse, D-35043 Marburg, Germany

## Abstract

The title compound, C_22_H_20_O, shows an approximately planar cyclo­hexenone ring [maximum deviation = 0.069 (4) Å], with a disordered position of the C=C bond [ratio = 0.71 (2)/0.29 (2)]. The benzene rings of the 4-methyl­benzyl­idene units, attached in the 2- and 6-positions to the cyclo­hexenone ring, are rotated in the same direction by 28.6 (4) and 22.4 (4)°, with respect to the mean plane of the cyclo­hexenone ring [fraction 0.71 (2); maximum deviation = 0.06 (3) Å]. In the crystal, mol­ecules are packed in the manner of a distorted hexa­gonal rod packing with their long axes all aligned along [201]. A number of C—H⋯π inter­actions stablize the crystal structure.

## Related literature

For background information to aldol condensation reactions in hetero- and homocyclic ketones, see: Abaee *et al.* (2007 ▶). For the crystal structure of the analogous compound with 4-meth­oxy­benzyl­idene substituents in the 2- and 6- positions on the cyclo­hexenone ring, see: Abaee *et al.* (2007 ▶). For other similar substituted cyclo­hexenone structures, see: Shi *et al.* (2008 ▶); Guo *et al.* (2008 ▶).
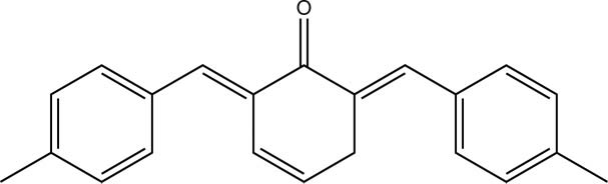


## Experimental

### 

#### Crystal data

C_22_H_20_O
*M* *_r_* = 300.38
Monoclinic, 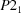

*a* = 10.7108 (14) Å
*b* = 7.2772 (7) Å
*c* = 11.4690 (14) Å
β = 114.366 (14)°
*V* = 814.32 (17) Å^3^
*Z* = 2
Mo *K*α radiation
μ = 0.07 mm^−1^
*T* = 193 K
0.50 × 0.24 × 0.15 mm

#### Data collection

Stoe IPDS image plate diffractometer
6110 measured reflections
1709 independent reflections
1219 reflections with *I* > 2σ(*I*)
*R*_int_ = 0.060

#### Refinement

*R*[*F* ^2^ > 2σ(*F* ^2^)] = 0.035
*wR*(*F* ^2^) = 0.071
*S* = 0.98
1709 reflections
212 parameters
3 restraints
H-atom parameters constrained
Δρ_max_ = 0.17 e Å^−3^
Δρ_min_ = −0.15 e Å^−3^

### 

Data collection: *EXPOSE* (Stoe & Cie, 1999 ▶); cell refinement: *CELL* (Stoe & Cie, 1999 ▶); data reduction: *INTEGRATE* (Stoe & Cie, 1999 ▶); program(s) used to solve structure: *SHELXS97* (Sheldrick, 2008 ▶); program(s) used to refine structure: *SHELXL97* (Sheldrick, 2008 ▶); molecular graphics: *DIAMOND* (Brandenburg, 2011 ▶); software used to prepare material for publication: *publCIF* (Westrip 2010 ▶).

## Supplementary Material

Crystal structure: contains datablock(s) I, global. DOI: 10.1107/S1600536811055632/su2352sup1.cif

Structure factors: contains datablock(s) I. DOI: 10.1107/S1600536811055632/su2352Isup2.hkl

Supplementary material file. DOI: 10.1107/S1600536811055632/su2352Isup3.cml

Additional supplementary materials:  crystallographic information; 3D view; checkCIF report

## Figures and Tables

**Table 1:** Hydrogen-bond geometry (Å, °) *Cg*1, *Cg*2 and *Cg*3 are the centroids of the C8–C11,C12a,C13, C1–C6 and C15–C20 rings, respectively.

*D*—H⋯*A*	*D*—H	H⋯*A*	*D*⋯*A*	*D*—H⋯*A*
C3—H3⋯*Cg*2^i^	0.95	2.78	3.538 (3)	137
C6—H6⋯*Cg*3^ii^	0.95	2.64	3.423 (3)	139
C16—H16⋯*Cg*3^iii^	0.95	2.85	3.496 (3)	126
C13—H13b⋯*Cg*1^ii^	0.99	2.89	3.642 (6)	134

Symmetry codes: (i) 
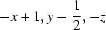
; (ii) 
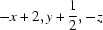
; (iii) 
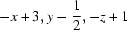
.

**Fig. 1.**
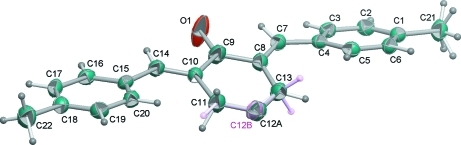
The molecular structure of the title molecule, with atom labels and 50% probability displacement ellipsoids. The less occupied (by 29%) disordered orientation of a part of the central ring is drawn in transparent red.

**Fig. 2.**
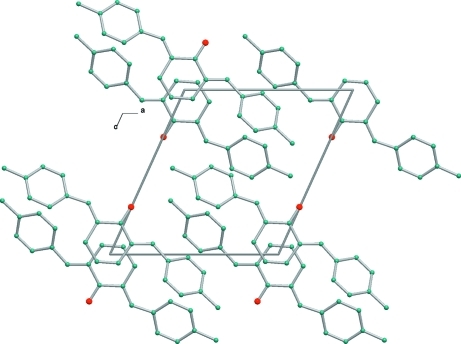
Packing diagram of the title compound viewed down the b axis, showing the parallel orientation of the molecules along the [201] direction; O-atoms red, H-atoms omitted.

**Fig. 3.**
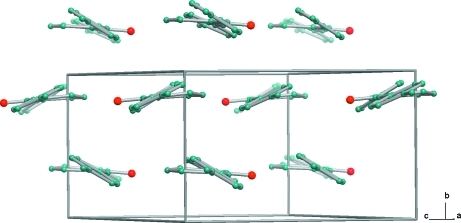
Arrangement of molecules according to a distorted hexagonal rod packing in projection along direction [201]; O-atoms red, H-atoms omitted.

## supplementary crystallographic information

### Comment

In continuation of our studies on aldol condensation reactions in hetero- and homocyclic ketones (Abaee *et al.*, 2007), we herein report on the synthesis and crystal structure of the title compound.

It crystallizes in the acentric space group P2_1_ but the molecule shows pseudosymmetry m (C_s_), with the mirror plane perpendicular to the main molecular plane. The C_s_ symmetry is broken by the central symmetry-less cyclohexenone ring, but a second orientation of the molecule generated by this mirror plane appears as an alternative disordered orientation in a ratio of 0.71 (2)/0.29 (2) (Fig. 1). A split atom model was refined [C12a/C12b: occupancies 0.71 (2)/(0.29 (2)] using restraints providing the same bond lengths for corresponding atom pairs, C11—C12a/C13—C12b and C11—C12b/C13—C12a, of 1.475 (5)/1.319 (5)Å. The strong anisotropy of the displacement parameters of the O atom may be associated to this disorder. In addition, one of the terminal methyl groups, C21, showed 1:1 disorder over two orientations [occupancy of 0.5 for each of the six H-atom postions].

A second pseudosymmetric mirror plane can be found in the main plane of the molecule, the realisation of which would lead to the centrosymmetric space group *P*2_1_/*m*. The clear inclination of both benzene rings [II (C1-C6) and III (C15-C20)] by 28.6 (4) and 22.4 (4)°, respectively, to the cyclohexenone ring I (O1,C8-C11,C12a,C13) rules out this possibility. Benzene rings II and III are inclined to one another by 8.66 (13)°.

In the presence of only one O atom besides 22 C and 20 H atoms, the absolute structure could not be determined, and from the synthesis the formation of a racemate is expected. In an analogous compound, with 4-methoxybenzylidene substituents in the 2- and 6- positions on the cyclohexenone ring (Abaee *et al.*, 2007), the benzene rings are rotated in opposite directions with respect to the central ring plane, while in the title compound the rotation is in the same direction (Fig. 1). In general the bond distances and angles are similar to those observed in analogues structures (Abaee *et al.*, 2007; Shi *et al.*, 2008; Guo *et al.*, 2008).

In the crystal, molecules pack in the manner of a distorted hexagonal rod packing with their long axes all aligned along the [201] direction (Figs. 2 and 3). The intermolecular contacts are reinforced by C–H···π interactions (Table 1).

### Experimental

A mixture of cyclohex-2-enone (193 µ*L*, 2 mmol), 4-methylbenzaldehyde (471 µ*L*, 4 mmol), triethylamine (1122 µ*L*, 8 mmol), and ZnBr_2_ (900 mg, 4 mmol) in 5 ml dry CH_2_Cl_2_ was stirred at room temperature under argon atmosphere for 10 h. The progress of the reaction was checked by TLC using a 1:8 mixture of EtOAc/hexane. At the end of the reaction, the mixture was diluted with CH_2_Cl_2_ and washed with brine. The organic layer was dried using Na_2_SO_4_ and concentrated under reduced pressure. The product obtained was isolated (540 mg, 90%) by column chromatography over silicagel using a 1:8 mixture of EtOAc/hexane. The solid product was recrystallized from EtOAc to give light-orange block-like crystals of the title compound.

### Refinement

For the disordered region of the central cyclohexenone ring, a split atom model was refined [C12a/C12b: occupancies 0.71 (2)/0.29 (2)] using restraints providing the same bond lengths for corresponding atom pairs: C11—C12a/C13—C12b = 1.475 (5)Å and C13—C12a/C11—C12b 1.319 (5) Å. The anisotropic displacement parameters of the split atoms, C12a and C12b, were set to be equal. In the final cycles of refinement, in the absence of significant anomalous scattering effects, 1386 Friedel pairs were merged and Δf " set to zero. All the H atoms could be located in a difference Fourier map. In the final cycles of refinement they were included in calculated positions and treated as riding atoms: C-H = 0.95 and 0.99 Å for CH and CH_3_ H-atoms, respectively, with U_iso_(H) = k × U_eq_(parent C-atom), where k = 1.5 for CH_3_ H-atoms and k = 1.2 for all other H-atoms.

### Crystal data

**Table:**

C_22_H_20_O	*F*(000) = 320
*M**_r_* = 300.38	*D*_x_ = 1.225 Mg m^−^^3^
Monoclinic, *P*2_1_	Mo *K*α radiation, λ = 0.71073 Å
Hall symbol: P 2yb	Cell parameters from 5168 reflections
*a* = 10.7108 (14) Å	θ = 2.0–25.8°
*b* = 7.2772 (7) Å	µ = 0.07 mm^−^^1^
*c* = 11.4690 (14) Å	*T* = 193 K
β = 114.366 (14)°	Block, light-orange
*V* = 814.32 (17) Å^3^	0.50 × 0.24 × 0.15 mm
*Z* = 2	

### Data collection

**Table:**

Stoe IPDS image plate diffractometer	1219 reflections with *I* > 2σ(*I*)
Radiation source: fine-focus sealed tube	*R*_int_ = 0.060
graphite	θ_max_ = 26.0°, θ_min_ = 2.0°
Detector resolution: 6.7 pixels mm^-1^	*h* = −13→13
φ–scans	*k* = −8→8
6110 measured reflections	*l* = −13→14
1709 independent reflections	

### Refinement

**Table:**

Refinement on *F*^2^	Primary atom site location: structure-invariant direct methods
Least-squares matrix: full	Secondary atom site location: difference Fourier map
*R*[*F*^2^ > 2σ(*F*^2^)] = 0.035	Hydrogen site location: difference Fourier map
*wR*(*F*^2^) = 0.071	H-atom parameters constrained
*S* = 0.98	*w* = 1/[σ^2^(*F*_o_^2^) + (0.030*P*)^2^] where *P* = (*F*_o_^2^ + 2*F*_c_^2^)/3
1709 reflections	(Δ/σ)_max_ < 0.001
212 parameters	Δρ_max_ = 0.17 e Å^−^^3^
3 restraints	Δρ_min_ = −0.15 e Å^−^^3^

### Special details

**Table:**

Geometry. All e.s.d.'s (except the e.s.d. in the dihedral angle between two l.s. planes) are estimated using the full covariance matrix. The cell e.s.d.'s are taken into account individually in the estimation of e.s.d.'s in distances, angles and torsion angles; correlations between e.s.d.'s in cell parameters are only used when they are defined by crystal symmetry. An approximate (isotropic) treatment of cell e.s.d.'s is used for estimating e.s.d.'s involving l.s. planes.

### Fractional atomic coordinates and isotropic or equivalent isotropic displacement parameters (Å^2^)

**Table:**

	*x*	*y*	*z*	*U*_iso_*/*U*_eq_	Occ. (<1)
O1	1.00222 (16)	0.8042 (5)	0.28700 (15)	0.0927 (11)	
C1	0.3618 (2)	0.8455 (4)	−0.22735 (19)	0.0337 (6)	
C2	0.3948 (2)	0.7517 (4)	−0.1120 (2)	0.0359 (6)	
H2	0.3241	0.6931	−0.0961	0.043*	
C3	0.5283 (2)	0.7427 (4)	−0.0206 (2)	0.0336 (6)	
H3	0.5473	0.6804	0.0577	0.040*	
C4	0.6368 (2)	0.8242 (4)	−0.04127 (19)	0.0310 (5)	
C5	0.6034 (2)	0.9148 (4)	−0.1573 (2)	0.0338 (6)	
H5	0.6739	0.9705	−0.1749	0.041*	
C6	0.4685 (2)	0.9248 (4)	−0.2476 (2)	0.0343 (6)	
H6	0.4489	0.9882	−0.3257	0.041*	
C7	0.7743 (2)	0.8148 (4)	0.0625 (2)	0.0344 (6)	
H7	0.7756	0.7966	0.1449	0.041*	
C8	0.8999 (2)	0.8275 (5)	0.0616 (2)	0.0354 (6)	
C9	1.0200 (2)	0.8195 (5)	0.1884 (2)	0.0460 (7)	
C10	1.1616 (2)	0.8318 (5)	0.19458 (18)	0.0323 (5)	
C11	1.1787 (2)	0.8531 (5)	0.07247 (19)	0.0428 (7)	
H11A	1.2201	0.9750	0.0734	0.051*	0.71 (2)
H11B	1.2450	0.7592	0.0710	0.051*	0.71 (2)
H11C	1.2680	0.8662	0.0748	0.051*	0.29 (2)
C12A	1.0537 (11)	0.837 (4)	−0.0473 (6)	0.041 (2)	0.71 (2)
H12A	1.0648	0.8253	−0.1250	0.049*	0.71 (2)
C12B	1.0710 (15)	0.854 (12)	−0.0385 (8)	0.041 (2)	0.29 (2)
H12B	1.0869	0.8655	−0.1137	0.049*	0.29 (2)
C13	0.9276 (2)	0.8378 (5)	−0.0544 (2)	0.0445 (6)	
H13A	0.8788	0.7335	−0.1099	0.053*	0.29 (2)
H13B	0.8832	0.9514	−0.1004	0.053*	0.29 (2)
H13C	0.8546	0.8343	−0.1373	0.053*	0.71 (2)
C14	1.2658 (2)	0.8164 (4)	0.3111 (2)	0.0351 (6)	
H14	1.2366	0.7976	0.3779	0.042*	
C15	1.4143 (2)	0.8230 (4)	0.35534 (19)	0.0312 (5)	
C16	1.4931 (2)	0.7558 (4)	0.4790 (2)	0.0342 (6)	
H16	1.4478	0.7056	0.5276	0.041*	
C17	1.6344 (2)	0.7606 (4)	0.5319 (2)	0.0382 (6)	
H17	1.6839	0.7136	0.6157	0.046*	
C18	1.7060 (2)	0.8332 (5)	0.4649 (2)	0.0372 (6)	
C19	1.6289 (2)	0.8998 (4)	0.3425 (2)	0.0371 (7)	
H19	1.6750	0.9490	0.2944	0.045*	
C20	1.4862 (2)	0.8969 (3)	0.2881 (2)	0.0350 (6)	
H20	1.4371	0.9455	0.2046	0.042*	
C21	0.2162 (2)	0.8587 (5)	−0.3252 (2)	0.0496 (8)	
H21A	0.1559	0.7939	−0.2938	0.074*	0.50
H21B	0.2086	0.8025	−0.4056	0.074*	0.50
H21C	0.1890	0.9881	−0.3401	0.074*	0.50
H21D	0.2131	0.9291	−0.3992	0.074*	0.50
H21E	0.1604	0.9205	−0.2875	0.074*	0.50
H21F	0.1800	0.7349	−0.3529	0.074*	0.50
C22	1.8600 (2)	0.8394 (6)	0.5234 (2)	0.0556 (8)	
H22A	1.8959	0.7480	0.5920	0.083*	
H22B	1.8909	0.9621	0.5587	0.083*	
H22C	1.8935	0.8122	0.4577	0.083*	

### Atomic displacement parameters (Å^2^)

**Table:**

	*U*^11^	*U*^22^	*U*^33^	*U*^12^	*U*^13^	*U*^23^
O1	0.0368 (9)	0.212 (3)	0.0306 (9)	0.0030 (17)	0.0155 (7)	0.0097 (17)
C1	0.0350 (13)	0.0347 (16)	0.0299 (12)	0.0045 (14)	0.0118 (10)	−0.0026 (14)
C2	0.0321 (13)	0.0397 (16)	0.0388 (14)	−0.0034 (12)	0.0176 (11)	−0.0027 (13)
C3	0.0386 (14)	0.0361 (16)	0.0296 (12)	0.0024 (13)	0.0176 (11)	0.0020 (12)
C4	0.0349 (12)	0.0289 (13)	0.0312 (11)	0.0045 (14)	0.0155 (9)	−0.0022 (15)
C5	0.0343 (13)	0.0342 (15)	0.0364 (13)	−0.0003 (12)	0.0182 (11)	−0.0004 (13)
C6	0.0394 (14)	0.0341 (15)	0.0301 (12)	0.0047 (12)	0.0149 (11)	0.0026 (11)
C7	0.0324 (12)	0.0400 (17)	0.0286 (11)	−0.0001 (13)	0.0105 (9)	−0.0028 (14)
C8	0.0299 (12)	0.0428 (14)	0.0316 (12)	0.0007 (15)	0.0107 (10)	0.0016 (15)
C9	0.0378 (13)	0.070 (2)	0.0324 (12)	0.0009 (16)	0.0169 (11)	−0.0034 (17)
C10	0.0311 (12)	0.0370 (14)	0.0277 (11)	0.0028 (15)	0.0110 (10)	−0.0013 (14)
C11	0.0362 (12)	0.062 (2)	0.0325 (12)	0.0033 (15)	0.0169 (10)	0.0044 (15)
C12A	0.041 (2)	0.054 (7)	0.0294 (12)	−0.002 (5)	0.0169 (14)	0.001 (3)
C12B	0.041 (2)	0.054 (7)	0.0294 (12)	−0.002 (5)	0.0169 (14)	0.001 (3)
C13	0.0358 (13)	0.0627 (18)	0.0334 (12)	0.0018 (16)	0.0126 (10)	−0.0047 (16)
C14	0.0315 (12)	0.0443 (17)	0.0306 (11)	−0.0003 (15)	0.0140 (10)	−0.0024 (15)
C15	0.0354 (12)	0.0321 (14)	0.0252 (11)	−0.0002 (14)	0.0117 (10)	−0.0059 (14)
C16	0.0386 (13)	0.0352 (16)	0.0319 (12)	−0.0023 (12)	0.0175 (11)	−0.0009 (12)
C17	0.0364 (14)	0.0416 (16)	0.0284 (12)	0.0007 (12)	0.0051 (11)	0.0001 (12)
C18	0.0318 (12)	0.0410 (15)	0.0361 (12)	0.0005 (15)	0.0112 (10)	−0.0045 (16)
C19	0.0361 (15)	0.0401 (17)	0.0393 (14)	−0.0032 (12)	0.0197 (12)	−0.0015 (13)
C20	0.0376 (14)	0.0359 (17)	0.0301 (12)	0.0001 (13)	0.0127 (11)	0.0018 (12)
C21	0.0363 (13)	0.066 (2)	0.0384 (13)	0.0014 (15)	0.0075 (11)	0.0007 (16)
C22	0.0354 (14)	0.072 (2)	0.0537 (16)	−0.003 (2)	0.0123 (12)	−0.004 (2)

### Geometric parameters (Å, °)

**Table:**

O1—C9	1.227 (3)	C12B—C13	1.475 (11)
C1—C6	1.383 (3)	C12B—H12B	0.9500
C1—C2	1.398 (3)	C13—H13A	0.9900
C1—C21	1.503 (3)	C13—H13B	0.9900
C2—C3	1.382 (3)	C13—H13C	0.9500
C2—H2	0.9500	C14—C15	1.458 (3)
C3—C4	1.409 (3)	C14—H14	0.9500
C3—H3	0.9500	C15—C20	1.403 (3)
C4—C5	1.393 (3)	C15—C16	1.406 (3)
C4—C7	1.465 (3)	C16—C17	1.379 (3)
C5—C6	1.390 (3)	C16—H16	0.9500
C5—H5	0.9500	C17—C18	1.395 (3)
C6—H6	0.9500	C17—H17	0.9500
C7—C8	1.352 (3)	C18—C19	1.390 (3)
C7—H7	0.9500	C18—C22	1.503 (3)
C8—C13	1.479 (3)	C19—C20	1.392 (3)
C8—C9	1.493 (3)	C19—H19	0.9500
C9—C10	1.491 (3)	C20—H20	0.9500
C10—C14	1.346 (3)	C21—H21A	0.9800
C10—C11	1.494 (3)	C21—H21B	0.9800
C11—C12B	1.319 (12)	C21—H21C	0.9800
C11—C12A	1.475 (9)	C21—H21D	0.9800
C11—H11A	0.9900	C21—H21E	0.9800
C11—H11B	0.9900	C21—H21F	0.9800
C11—H11C	0.9500	C22—H22A	0.9800
C12A—C13	1.319 (10)	C22—H22B	0.9800
C12A—H12A	0.9500	C22—H22C	0.9800
			
C6—C1—C2	117.35 (19)	C12A—C13—H13B	106.9
C6—C1—C21	121.5 (2)	C12B—C13—H13B	104.3
C2—C1—C21	121.1 (2)	C8—C13—H13B	106.9
C3—C2—C1	121.3 (2)	H13A—C13—H13B	106.7
C3—C2—H2	119.4	C12A—C13—H13C	117.4
C1—C2—H2	119.4	C12B—C13—H13C	120.7
C2—C3—C4	121.3 (2)	C8—C13—H13C	120.7
C2—C3—H3	119.4	H13A—C13—H13C	48.5
C4—C3—H3	119.4	H13B—C13—H13C	58.2
C5—C4—C3	117.09 (19)	C10—C14—C15	133.0 (2)
C5—C4—C7	125.0 (2)	C10—C14—H14	113.5
C3—C4—C7	117.8 (2)	C15—C14—H14	113.5
C6—C5—C4	121.0 (2)	C20—C15—C16	116.78 (19)
C6—C5—H5	119.5	C20—C15—C14	126.1 (2)
C4—C5—H5	119.5	C16—C15—C14	117.1 (2)
C1—C6—C5	122.0 (2)	C17—C16—C15	121.9 (2)
C1—C6—H6	119.0	C17—C16—H16	119.1
C5—C6—H6	119.0	C15—C16—H16	119.1
C8—C7—C4	131.4 (2)	C16—C17—C18	121.4 (2)
C8—C7—H7	114.3	C16—C17—H17	119.3
C4—C7—H7	114.3	C18—C17—H17	119.3
C7—C8—C13	125.34 (19)	C19—C18—C17	117.1 (2)
C7—C8—C9	116.8 (2)	C19—C18—C22	121.8 (2)
C13—C8—C9	117.79 (19)	C17—C18—C22	121.1 (2)
O1—C9—C10	120.20 (19)	C18—C19—C20	122.2 (2)
O1—C9—C8	120.1 (2)	C18—C19—H19	118.9
C10—C9—C8	119.67 (19)	C20—C19—H19	118.9
C14—C10—C9	116.93 (19)	C19—C20—C15	120.6 (2)
C14—C10—C11	124.51 (19)	C19—C20—H20	119.7
C9—C10—C11	118.53 (18)	C15—C20—H20	119.7
C12B—C11—C12A	6(4)	C1—C21—H21A	109.5
C12B—C11—C10	120.5 (4)	C1—C21—H21B	109.5
C12A—C11—C10	116.7 (3)	H21A—C21—H21B	109.5
C12B—C11—H11A	102.5	C1—C21—H21C	109.5
C12A—C11—H11A	108.1	H21A—C21—H21C	109.5
C10—C11—H11A	108.1	H21B—C21—H21C	109.5
C12B—C11—H11B	109.5	C1—C21—H21D	109.5
C12A—C11—H11B	108.1	H21A—C21—H21D	141.1
C10—C11—H11B	108.1	H21B—C21—H21D	56.3
H11A—C11—H11B	107.3	H21C—C21—H21D	56.3
C12B—C11—H11C	119.7	C1—C21—H21E	109.5
C12A—C11—H11C	123.4	H21A—C21—H21E	56.3
C10—C11—H11C	119.7	H21B—C21—H21E	141.1
H11A—C11—H11C	57.9	H21C—C21—H21E	56.3
H11B—C11—H11C	49.5	H21D—C21—H21E	109.5
C13—C12A—C11	124.8 (4)	C1—C21—H21F	109.5
C13—C12A—H12A	117.6	H21A—C21—H21F	56.3
C11—C12A—H12A	117.6	H21B—C21—H21F	56.3
C11—C12B—C13	124.8 (6)	H21C—C21—H21F	141.1
C11—C12B—H12B	117.6	H21D—C21—H21F	109.5
C13—C12B—H12B	117.6	H21E—C21—H21F	109.5
C12A—C13—C12B	6(4)	C18—C22—H22A	109.5
C12A—C13—C8	121.6 (3)	C18—C22—H22B	109.5
C12B—C13—C8	118.5 (3)	H22A—C22—H22B	109.5
C12A—C13—H13A	106.9	C18—C22—H22C	109.5
C12B—C13—H13A	112.7	H22A—C22—H22C	109.5
C8—C13—H13A	106.9	H22B—C22—H22C	109.5
			
C6—C1—C2—C3	1.5 (4)	C12B—C11—C12A—C13	−121 (34)
C21—C1—C2—C3	−178.6 (3)	C10—C11—C12A—C13	12 (3)
C1—C2—C3—C4	−1.4 (4)	C12A—C11—C12B—C13	50 (25)
C2—C3—C4—C5	0.3 (4)	C10—C11—C12B—C13	1(9)
C2—C3—C4—C7	177.6 (2)	C11—C12A—C13—C12B	50 (25)
C3—C4—C5—C6	0.5 (4)	C11—C12A—C13—C8	−9(3)
C7—C4—C5—C6	−176.5 (2)	C11—C12B—C13—C12A	−121 (34)
C2—C1—C6—C5	−0.7 (4)	C11—C12B—C13—C8	3(9)
C21—C1—C6—C5	179.5 (3)	C7—C8—C13—C12A	−175.2 (17)
C4—C5—C6—C1	−0.4 (4)	C9—C8—C13—C12A	1.1 (17)
C5—C4—C7—C8	−25.5 (5)	C7—C8—C13—C12B	179 (4)
C3—C4—C7—C8	157.5 (3)	C9—C8—C13—C12B	−5(4)
C4—C7—C8—C13	−6.2 (6)	C9—C10—C14—C15	−179.3 (3)
C4—C7—C8—C9	177.4 (3)	C11—C10—C14—C15	3.0 (6)
C7—C8—C9—O1	−0.7 (5)	C10—C14—C15—C20	18.6 (6)
C13—C8—C9—O1	−177.4 (4)	C10—C14—C15—C16	−164.2 (3)
C7—C8—C9—C10	179.7 (3)	C20—C15—C16—C17	−0.5 (4)
C13—C8—C9—C10	3.1 (5)	C14—C15—C16—C17	−178.0 (3)
O1—C9—C10—C14	3.0 (5)	C15—C16—C17—C18	0.1 (4)
C8—C9—C10—C14	−177.4 (3)	C16—C17—C18—C19	−0.2 (4)
O1—C9—C10—C11	−179.1 (4)	C16—C17—C18—C22	179.5 (3)
C8—C9—C10—C11	0.4 (5)	C17—C18—C19—C20	0.6 (4)
C14—C10—C11—C12B	175 (4)	C22—C18—C19—C20	−179.1 (3)
C9—C10—C11—C12B	−3(4)	C18—C19—C20—C15	−1.0 (4)
C14—C10—C11—C12A	170.2 (14)	C16—C15—C20—C19	0.9 (4)
C9—C10—C11—C12A	−7.5 (15)	C14—C15—C20—C19	178.1 (3)

### Hydrogen-bond geometry (Å, °)

**Table:**

Cg1, Cg2 and Cg3 are the centroids of the C8–C11,C12a,C13, C1–C6 and C15–C20 rings, respectively.

**Table:**

*D*—H···*A*	*D*—H	H···*A*	*D*···*A*	*D*—H···*A*
C3—H3···Cg2^i^	0.95	2.78	3.538 (3)	137
C6—H6···Cg3^ii^	0.95	2.64	3.423 (3)	139
C16—H16···Cg3^iii^	0.95	2.85	3.496 (3)	126
C13—H13b···Cg1^ii^	0.99	2.89	3.642 (6)	134

Symmetry codes: (i) −*x*+1, *y*−1/2, −*z*; (ii) −*x*+2, *y*+1/2, −*z*; (iii) −*x*+3, *y*−1/2, −*z*+1.
